# Human papillomavirus infection in women with and without cervical cancer in Ibadan, Nigeria

**DOI:** 10.1186/1750-9378-5-24

**Published:** 2010-12-03

**Authors:** Clement Okolo, Silvia Franceschi, Isaac Adewole, Jaiye O Thomas, Michele Follen, Peter JF Snijders, Chris JLM Meijer, Gary M Clifford

**Affiliations:** 1 College of Medicine, University of Ibadan, Ibadan, Nigeria; 2 International Agency for Research on Cancer, Lyon, France; 3 Drexel University College of Medicine, Philadelphia, Pennsylvania, USA; 4 VU University Medical Center, Amsterdam, The Netherlands

## Abstract

**Background:**

Concerns have been raised that the proportion of cervical cancer preventable by human papillomavirus (HPV) 16/18 vaccines might be lower in sub-Saharan Africa than elsewhere.

**Method:**

In order to study the relative carcinogenicity of HPV types in Nigeria, as well as to estimate the vaccine-preventable proportion of invasive cervical cancer (ICC) in the country, we compared HPV type prevalence among 932 women from the general population of Ibadan, Nigeria, with that among a series of 75 ICC cases diagnosed in the same city. For all samples, a GP5+/6+ PCR based assay was used for the detection of 44 genital HPV types.

**Results:**

In the general population, 245 (26.3%, 95% confidence interval (CI) 23.5% - 29.2%) women were HPV-positive, among whom the prevalence of HPV35 and HPV16 were equally frequent (12.2%, 95% CI 8.4% - 17.0%). In ICC, however, HPV16 predominated strongly (67.6% of 68 HPV-positive cases), with the next most common types being 18 (10.3%, 95% CI 4.2% - 20.1%), 35, 45 and 56 (each 5.9%, 95% CI 1.6% - 14.4%). Comparing among HPV-positive women only, HPV16 and 18 were over-represented in ICC *versus *the general population (prevalence ratios 5.52, 95% CI 3.7 - 8.3 and 1.4, 95% CI 0.6 - 3.3, respectively). Other high-risk HPV types, as well as low-risk and multiple HPV infections were less common in HPV-positive women with ICC than from the general population.

**Conclusions:**

Our study confirms that in Nigeria, as elsewhere, women infected with HPV16 and 18 are at higher risk of developing ICC than those infected with other high-risk types, and that current HPV16/18 vaccines have enormous potential to reduce cervical cancer in the region.

## Background

Cervical cancer incidence in sub-Saharan Africa is among the highest in the world [1], as is the prevalence of cervical human papillomavirus (HPV) infection [2]. In a pooled analysis of the International Agency for Research on Cancer (IARC) HPV Prevalence Surveys including 13 populations worldwide, the highest HPV prevalence was observed in Nigeria [2,3].

HPV16 prevalence was also the highest in Nigeria. However, in relative terms, HPV-positive women in Nigeria were less likely to be infected with HPV16 and more likely to be infected with other high-risk HPV types, in particular HPV35, but also HPV45, 52, 56 and 58 [2]. This, and similar findings from other sub-Saharan African populations [4], raised concerns that the proportion of HPV disease preventable by HPV16/18 vaccines might be lower in sub-Saharan Africa than elsewhere. However, high-risk HPV types differ in their risk for progression from infection to invasive cervical cancer (ICC) [5,6]. Thus, HPV type distribution at the population level is not representative of that in ICC, for which no data from Nigeria exist to date.

In order to study the relative carcinogenicity of HPV types in Nigeria, as well as to obtain a more robust measure of the vaccine-preventable proportion of ICC in the country, we compared HPV type-specific prevalence in the previously published IARC prevalence study with that among a newly collected series of ICC cases diagnosed in the same city and tested for HPV using the same protocol.

## Methods

Between April 1999 and April 2000, a previously published study of HPV prevalence was carried out among a representative sample of women living in Ibadan, the third largest city in Nigeria, according to the standardized protocol of the IARC HPV Prevalence Surveys [3], which was approved by both the IARC and local ethical review committees. In summary, all women aged 15 years or older living in Idikan, a district of Ibadan, were visited at their homes and invited to join the study, with the aim to enroll approximately 100 women in each 5-year age group. Of the invited women, 1,203 signed an informed consent form, underwent a pelvic examination and cervical screening by two methods: Papanicolau smear and visual inspection with acetic acid. In addition, samples of cervical exfoliated cells in phosphate buffer saline were collected for HPV testing.

Tumour biopsies, formalin fixed and embedded in paraffin, were retrieved from the pathology archives for all women diagnosed with histologically confirmed ICC (n = 102) at the College of Medicine, University of Ibadan, Nigeria, between 2004 and 2006.

HPV testing of cervical samples from both the general population and ICC cases were performed at the VU University, Amsterdam, the Netherlands. Tumour biopsies were sectioned using a 'sandwich' approach: inner sections were destined for HPV testing and outer sections for histological confirmation. One or more five μM sections representing approximately 1 cm^2 ^of tissue were predigested with Proteinase K after which DNA was extracted using magnetic beads (Macherey-Nagel, Germany). For all samples, a general primer GP5+/6+-mediated PCR was used for the detection of 44 genital HPV types, after screening for adequate DNA by PCR with beta-globin gene-specific primers. Subsequent HPV typing was performed by reverse-line blot hybridization of PCR products.

Prevalence ratios and corresponding 95% confidence intervals (CI) were used to compare the relative frequency of HPV types in HPV-positive women with ICC with that among HPV-positive women from the general population.

## Results

For the general population sample, type-specific HPV prevalence was evaluated among 932 women with valid HPV results, among whom 844 and 68 had normal and abnormal (positive on PAP and/or VIA) cervical findings, respectively. Overall HPV prevalence was 26.3% (95% CI 23.5 - 29.2) (n = 245). HPV16 and 35 were equally common (n = 30), followed by HPV31 (n = 27), 56 (n = 22) and 58 (n = 21). Two women with abnormal cervical findings were subsequently histologically confirmed with *in situ *(HPV18/42/52/81/83) or invasive (HPV82) cervical carcinoma.

For ICC, after exclusion of biopsies without any histological evidence of tumour (n = 4) or inadequate DNA for HPV testing (n = 23), type-specific HPV prevalence was evaluated among 75 histologically confirmed ICC cases. They included 62 squamous-cell, 11 adeno-, and 2 small cell carcinomas. The median age of women with ICC was 55 years (range: 31-80 years). Overall HPV prevalence was 90.7% (95% CI 81.7 - 96.2). In seven ICC, including 3 squamous-cell and four adenocarcinoma (ADC), no HPV DNA could be detected. The five most commonly detected types in ICC were HPV16 (n = 46), 18 (n = 7), 35, 45 and 56 (n = 4 each). The HPV types among the 7 HPV-positive ADC were four HPV16 and one each of HPV18, 35 and 56. One ICC was positive for a low-risk type only (HPV30).

The comparison of type-specific HPV prevalence among the 68 women with HPV-positive ICC and 245 HPV-positive women from the general population is shown in Table 1. HPV16 was significantly over-represented in HPV-positive women with ICC (prevalence ratio = 5.5, 95% CI 3.7 - 8.3). HPV18 was the only other type to be over-represented in ICC, although not significantly so (1.4, 95% CI 0.6 - 3.3). Other individual high-risk HPV types were less common in HPV-positive women with ICC than those from the general population. In particular, HPV31, 52 and 58 were common in the general population, found in 11.0%, 5.3% and 8.6% of HPV-positive women, respectively, but significantly less prevalent in ICC. Low-risk and multiple HPV infections were also much less common in HPV-positive women with ICC than those from the general population. HPV16 and/or 18 were detected in a total of 77.9% of HPV-positive ICC, and HPV types 16, 18, 31, 33 and 45 in a total of 85.2% (Table 1).

**Table 1:** Comparison of HPV type-specific prevalence among HPV-positive women from the general population and with ICC

HPV type	General Population (N = 245)	ICC (N = 68)	
			
	n (%, 95% CI)	n (%, 95% CI)	Prevalence ratio (95% CI)
16	30 (12.2, 8.4 - 17.0)	46 (67.6, 55.2 - 78.5)	5.5 (3.7-8.3)
18	18 (7.3, 4.4 - 11.4)	7 (10.3, 4.2 - 20.1)	1.4 (0.6-3.3)
45	19 (7.8, 4.7 - 1.8)	4 (5.9, 1.6 - 14.4)	0.8 (0.3-2.2)
35	30 (12.2, 8.4 - 17.0)	4 (5.9, 1.6 - 14.4)	0.5 (0.2-1.3)
56	22 (9.0, 5.1 - 12.3)	4 (5.9, 1.6 - 14.4)	0.7 (0.2-1.9)
51	14 (5.7, 3.2 - 9.4)	3 (4.4, 0.9 - 12.4)	0.8 (0.2-2.7)
31	27 (11.0, 7.4 - 15.6)	1 (1.5, 0.1 - 7.9)	0.1 (0.0-0.7)
33	5 (2.0, 0.7 - 4.7)	1 (1.5, 0.1 - 7.9)	0.7 (0.0-4.1)
39	6 (2.4, 0.9 - 5.3)	1 (1.5, 0.1 - 7.9)	0.6 (0.0-3.4)
73	4 (1.6, 0.4 - 4.1)	1 (1.5, 0.1 - 7.9)	0.9 (0.0-5.2)
52	13 (5.3, 2.9 - 8.9)	0 (0.0, 0.0 - 5.2)	0.0 (0.0-0.99)
58	21 (8.6, 5.4 -12.8)	0 (0.0, 0.0 - 5.2)	0.0 (0.0-0.6)
68	2 (0.8, 0.1 - 2.8)	0 (0.0, 0.0 - 5.2)	0.0 (0.0-6.8)
82	4 (1.6, 0.4 - 4.1)^a^	0 (0.0, 0.0 - 5.2)	0.0 (0.0-3.4)
Any high-risk	169 (69.0, 62.8 - 74.7)	67 (98.5, 92.1 - 99.9)	1.4 (1.1-1.9)
			
Any low-risk	110 (44.9, 38.6 - 51.4)	2 (2.9, 0.4 - 10.2) ^b^	0.1 (0.0-0.2)
			
Any multiple	68 (27.8, 22.2 - 33.8)	5 (7.4, 2.4 - 16.3) ^c^	0.3 (0.1-0.6)
			
16/18	47 (19.2, 14.4 - 24.7)	53 (77.9, 66.2 - 87.1)	4.1 (2.8-5.8)
16/18/31/33/45	90 (36.7, 30.7 - 43.1)	58 (85.2, 74.6 - 92.7)	2.3 (1.7-3.2)

CI = confidence interval; HPV = human papillomavirus; ICC = invasive cervical cancer

^a ^one woman subsequently confirmed with ICC.^b^1 ICC was positive for a low-risk type only (HPV30). ^c^Including 16/35, 16/45, 16/51, 16/53/56, 16/56, for which the individual types are counted separately.

## Discussion

We previously reported that HPV-positive women from the general population in Nigeria were less likely to be infected with HPV16 and more likely to be infected with other high-risk HPV types, in particular HPV35 [2]. Indeed, relatively high prevalence of HPV35 in comparison to HPV16 has also been observed from other sub-Saharan African populations including Guinea [7], Mozambique [4], Kenya [8] Gambia [9,10], and Zimbabwe [11], whereas HPV35 is four- to five-fold less prevalent than HPV16 in population-based surveys from Europe and the Americas [2,12,13]. These findings for HPV35, but also other high-risk types, raised concerns that the relevance of HPV16/18 vaccines to cervical cancer prevention might be lower in Nigeria or in sub-Saharan Africa in general.

Reassuringly, however, we confirmed that HPV16 and 18 are the two most frequently detected types among ICC in Nigeria, and that they account for 78% of HPV-positive ICC, which is very similar to the proportion estimated in other world regions by recently published large meta-analyses [14] and case series [15]. This finding adds to the picture of a relatively homogeneous type-distribution in ICC around the world [14]. Our cross-sectional findings of an enrichment of these two types in ICC are also consistent with other cross-sectional data from Sub-Saharan Africa [7,16] as well as with evidence from prospective studies that show that women infected with HPV16 and HPV18 are at significantly higher risk of developing CIN3 and cancer in comparison to women infected with other high-risk types [5,6,17].

Despite being as common as HPV16 in the general population, HPV35 accounted for only 5.9% of ICC. This figure is compatible with estimates of 5.0% (95% CI: 3.3% - 7.1%) and 4.1% (95% CI: 2.3% - 7.4%) among two recently published studies including 544 and 1,668 African ICC cases, respectively, assessed for this type [14], but is higher than that in ICC from other world regions (1% - 3%) [14,15]. Thus, although the relative carcinogenicity of HPV35 is clearly less than HPV16, it does still account for a substantial fraction of ICC in sub-Saharan African populations and should be considered for new generation vaccines.

A relatively high importance of HPV45 (5.3%) and of HPV51 (4.0%) in Nigerian ICC is also consistent with that observed in other sub-Saharan Africa ICC in comparison to other world regions [14,15]. On the other hand, HPV31 was considerably less common in ICC than in the general population, as seen in previous studies from Guinea [7] and Mozambique [16], suggesting that in sub-Saharan African populations, it has only a relatively weak carcinogenic potential in comparison to other types. Of note, one ICC case was positive for HPV30 only, and another for HPV82 only, both of which are classified as "possibly carcinogenic to humans" by IARC based upon their phylogenetic similarity to other high-risk types [18]. Interestingly, HPV30 was reported in only one out of 2,851 worldwide ICC (0.04%) in a recent meta-analysis [14], a HPV30 single infection from an ICC case in Guinea, also in Western Africa [7].

## Conclusions

In summary, our study confirms that current HPV vaccines have enormous potential to reduce cervical cancer in Nigeria, where annual ICC incidence is estimated to be 19.3 cases/100,000 women [1]. An efficacious vaccine against HPV16/18 is likely to prevent more than 70% of the 14,550 new ICC cases and 9,659 ICC deaths that are estimated to occur in Nigeria every year [1]. If vaccines also offer full cross-protection against HPV31, 33 and 45 [19,20], then the prevented proportion with existing HPV16/18 vaccines could be as high as 85%.

## List of Abbreviations

ADC: adenocarcinoma; CI: confidence interval; HPV: human papillomavirus; IARC: International Agency for Research on Cancer; ICC: invasive cervical cancer; OR: odds ratio.

## Competing interests

The authors declare that they have no competing interests.

## Authors' contributions

GC conceived the study, performed statistical analysis, and drafted the manuscript. MF, IA and SF participated in the design and coordination of the study and helped to draft the manuscript. CO and JT were responsible for sample and data acquisition. PS and CM were responsible for all laboratory analyses. All authors contributed to data interpretation and read and approved the final manuscript.
